# Effects of lifestyle modification after breast cancer treatment: a systematic review protocol

**DOI:** 10.1186/2046-4053-3-72

**Published:** 2014-07-05

**Authors:** Maicon Falavigna, Karine Margarites Lima, Juliana Giacomazzi, Diego d’Avila Paskulin, Luciano Serpa Hammes, Rodrigo Antonini Ribeiro, Daniela Dornelles Rosa

**Affiliations:** 1 Institute for Education and Research, Hospital Moinhos de Vento, Rua Ramiro Barcellos 910, Porto Alegre 90035-001, Brazil; 2 Department of Clinical Epidemiology and Biostatistics, McMaster University, 1280 Main Street West, Hamilton L8S 4L8, Canada; 3 Postgraduate Program in Epidemiology, Universidade Federal do Rio Grande do Sul, Rua Ramiro Barcellos 2400, Porto Alegre 90035-003, Brazil; 4 Molecular Biology Laboratory, Santa Casa de Misericórdia de Porto Alegre, Rua Professor Annes Dias 295, Porto Alegre 90035-001, Brazil; 5 Oncology Unit, Hospital Moinhos de Vento, Hospital Moinhos de Vento, Rua Ramiro Barcellos 910, Porto Alegre 90035-001, Brazil

**Keywords:** Breast cancer, Lifestyle interventions, Exercise, Diet, Physical activity, Systematic review, Adjuvant chemotherapy

## Abstract

**Background:**

There is no consensus in the literature regarding the effectiveness of lifestyle modification interventions, including recommendations about specific diet or exercise program for patients with breast cancer. Diet interventions and regular physical activity may reduce the risk of breast cancer and its recurrence. The primary aim of our study is to evaluate the effects of different lifestyle modification interventions (diet and physical activity) in the survival of patients with stages I to III breast cancer after treatment.

**Methods/design:**

This review will be conducted according to the Cochrane Handbook for Systematic Reviews of Intervention and will be reported following the PRISMA statement recommendations. CENTRAL, MEDLINE and EMBASE databases will be searched for peer-reviewed literature. Randomized controlled trials of diet, exercise, or both, compared with usual care, after treatment of breast cancer stage I to III will be included in the systematic review. Two authors will independently screen titles and abstracts of studies for potential eligibility. Data will be combined using random-effect meta-analysis models with restricted maximum-likelihood as variance estimator, and will be presented as relative risk or standardized mean difference with 95% CI. The quality of evidence will be assessed using the Grading of Recommendations Assessment, Development and Evaluation (GRADE) framework and summary of findings tables will be presented for patient important outcomes.

**Discussion:**

Our study may improve the current understanding of the role that lifestyle-modifiable factors can play in saving or prolonging the lives of women who have been treated for breast cancer, and also on modifying their quality of life.

**Systematic review registration:**

The review has been registered with PROSPERO (registration number CRD42014008743).

## Background

High body mass index (BMI) is well established as a risk factor for the development of breast cancer, especially for post-menopausal women [1,2]. More than half of women diagnosed with breast cancer experience an increase in body weight associated with chemotherapy and treatment-related menopause [3]. Additionally, there is evidence that overweight or obese women and women with weight gain after diagnosis have an increased risk of disease recurrence and death compared to eutrophic women [4-8]. Women with a high body mass index have double the risk of five-year breast cancer recurrence and a 60% increased risk of death over 10 years in comparison to women with a normal BMI [6].

Dietary energy restriction reduces body weight, promoting a positive effect on psychological wellbeing in obese women and breast cancer survivors [9-11]. Weight loss interventions that reduce the dietary intake of fat to between 18 and 25% of total calories can also evoke a significant reduction in serum estrogen levels in pre- and post-menopausal women [12]. A diet rich in vegetables and fruits may decrease the risk of breast cancer and a diet high in total fat may increase the risk [13]. However, evidence of an association between a diet high in vegetables and fruit and low in total fat, and prevention of cancer progression has been conflicting in epidemiological studies [8,14-21].

Regular physical activity can help to control body weight and is known to reduce the risk of breast cancer [22-25]. Additional studies also suggest that it can halve the risk of death in breast cancer patients [26,27]. Regular physical activity can also have a positive effect on psychological health status and quality of life in breast cancer survivors that could enhance immune function through the normalization of stress hormone levels [28-30].

There is no consensus in the literature regarding the effectiveness of lifestyle modification interventions, including recommendations about a specific diet or exercise program for patients with breast cancer. The results of observational studies evaluating lifestyle modification and breast cancer recurrence have been mixed. In addition, the randomized clinical trials that have been published are inconclusive [31,32] and there are no recent systematic reviews assessing this topic. Therefore, the primary aim of our study is to evaluate the effects of different lifestyle modification interventions (diet and physical activity) in the survival and disease-free survival of patients with stages I to III breast cancer after treatment.

## Methods/design

We will conduct this review according to the Cochrane Handbook for Systematic Reviews of Intervention [33] and we will report data following the PRISMA statement recommendations [34].We will assess the quality of evidence for each outcome according to Grading of Recommendations Assessment, Development and Evaluation (GRADE) framework [35,36]. The review has been registered with PROSPERO (registration number: CRD42014008743).

### Inclusion criteria

The type of studies included will be randomized controlled trials of diet, exercise, or both, compared to usual care, after treatment of breast cancer stage I to III. The participants to be included will be women with invasive breast cancer, stage I to III, who were treated with curative intent in the previous five years, with no evidence of disease recurrence. The types of interventions considered will be as follows: (1) dietary advice delivered through group meetings, by telephone calls, or by mail correspondence; (2) individualized dietary counseling; (3) prescription of a specific diet, such as a calorie-restricted diet; (4) any type of exercise counseling that encouraged women to engage in regular recreational exercises, such as walking, jogging or sports; and (5) structured or individualized exercise programs or interventions in which women participated in supervised exercise sessions.

### Types of outcomes

The primary outcomes of the systematic review are overall survival and disease-free survival (five years post treatment and at the study maximum follow-up period for both outcomes).

We will also evaluate the following secondary outcomes: (1) diet and exercise related modifiable risk factors (weight, BMI, waist-hip ratio and body fat); (2) mediators and other metabolic factors potentially associated with breast cancer (adiponectin, leptin, IGF-1, IGFBP1, IGFBP2, insulin, C-peptide, HOMA-IR, estradiol, testosterone and SHBG); (3) quality of life; and (4) adverse events (such as exercise-induced injuries or side effects of very low-calorie diets).

### Search strategy and sources

The following electronic databases will be searched for peer-reviewed literature: Cochrane Database of Systematic Reviews, Cochrane Central Register of Controlled Trials (CENTRAL), MEDLINE, and EMBASE. The search strategy was developed based on search strategies from published filters and systematic reviews [37-39], and is detailed in Additional file 1.

In addition, we will perform an electronic search for ongoing studies at ClinicalTrials.gov and hand searches of reference lists of included articles and of the proceedings of the following major conferences: American Society of Clinical Oncology (2010 to 2014), San Antonio Breast Cancer Symposium (2010 to 2014), European Society of Medical Oncology (2010 to 2014), Society for Integrative Oncology (2010 to 2014), American Association for Cancer Research (AACR) (2010 to 2014), and World Cancer Research Fund International (2010 to 2014).

### Data collection and analysis

#### Study selection

Two authors will independently screen titles and abstracts of studies for potential eligibility. Full texts of potentially eligible studies will be retrieved and two authors will independently apply inclusion criteria to identify relevant studies to be included in the review. Disagreement will be resolved through discussion; if necessary, a third reviewer will be involved. We will provide a table with characteristics of included studies, and another table of excluded studies with reasons for their exclusion, in the final review.

#### Data extraction and management

Two reviewers will independently extract data using a standardized form. The following data will be abstracted: (1) characteristics of trial participants (age, ethnicity, BMI, weight, body fat, waist-hip ratio, breast cancer stage, treatment, and breast cancer biological subtype); (2) type of intervention (such as type of diet or exercise program); (3) outcome measures and their definition according to individual studies; and (4) methodological quality of individual studies, according to the Cochrane Handbook for Systematic Reviews [33].

Disagreement will be resolved through discussion. When quantitative data is not reported, approximate values will be estimate from the figures or calculated from proportions.

#### Quality assessment

The risk of bias of all eligible studies will be assessed independently by two reviewers using the Cochrane Collaboration’s Risk of Bias tool [33]. Disagreement will be resolved through discussion. Overall quality of evidence will be assessed using GRADE by a GRADE working group member and will checked by a second reviewer [35,36].

#### Data synthesis and presentation

Data will be combined using random-effect meta-analysis models, with restricted maximum-likelihood (REML) variance estimator and presented as relative risks (RR) or standardized mean difference (SMD) with 95% confidence intervals (CI). All analyses will be performed using the R software, version 3.0.2 (R: A Language and Environment for Statistical Computing, Vienna, Austria); packages ‘meta’ version 3.0-1 (meta: Meta-Analysis with R. R package version) and ‘metafor’ version 1.9-1 (Conducting meta-analyses in R with the metafor package) [40,41]. GRADE summary of findings tables will be presented for the primary outcomes [42,43].

#### Heterogeneity

We will assess statistical heterogeneity in each meta-analysis using the I^2^ statistics. We will regard heterogeneity as substantial if the I^2^ is greater than 50%. Heterogeneity will be explored through pre-specified subgroup and sensitivity analysis as presented below.

#### Publication bias

If there are 10 or more studies in the meta-analysis we will investigate publication bias using funnel plots and Egger`s test [44]. If asymmetry is suggested by a visual assessment, we will perform exploratory analyses to investigate and adjust it (trim and fill analysis) [45].

#### Missing data

For included studies, we will note levels of attrition. We will explore the impact of including studies with high levels of missing data in the overall assessment of treatment effect by using sensitivity analysis.

For all outcomes, we will carry out analyses, as far as possible, on an intention-to-treat basis, in that we will attempt to include all participants randomized to each group in the analyses, and all participants will be analyzed in the group to which they were allocated, regardless of whether or not they received the allocated intervention. The denominator for each outcome in each trial will be the number randomized minus the number of participants whose outcomes are known to be missing.

#### Sensitivity analysis

In order to identify potential sources of heterogeneity, we will perform the following subgroup analysis: (1) type of intervention (diet, exercise, or both); (2) breast cancer treatment (adjuvant versus neoadjuvant); (3) tumor stage (according to AJCC) [46]; (4) subtypes of breast cancer according to immunohistochemistry (positivity for HR and/or HER2); (5) Mean follow-up period (≥24 months) and (6) risk of bias of included studies). Additionally, meta-regression will be performed according to the mean follow-up period of the included studies.

## Discussion

The results of this study may improve our understanding of the role that lifestyle-modifiable factors can play in saving or prolonging the lives of women who have been treated for breast cancer, and also on modifying their quality of life. Additionally, this study will provide evidence that may be used for the development of recommendations in guidelines of breast cancer treatment.

## Abbreviations

AJCC: American Joint Committee on Cancer; BMI: Body mass index; HER2: Human epidermal growth factor receptor 2; HOMA-IR: Homeostasis model assessment-estimated insulin resistance; HR: Hormone receptor; IGF1: Insulin-like growth factor 1; IGFBP1: Insulin-like growth factor-binding protein 1; IGFBP2: Insulin-like growth factor-binding protein 2; SHBG: Sex hormone-binding globulin.

## Competing interests

The authors declare that they have no competing interests.

## Authors’ contributions

DDR conceived the study and gave final approval for the protocol. MF selected methods for use, developed the search strategy and drafted the protocol. KML collaborated in the design of the study, reviewed and edited the protocol. JG collaborated in the design of the study, reviewed and edited the protocol. DDP collaborated in the design of the study, reviewed and edited the protocol. LSH collaborated in the design of the study, reviewed and edited the protocol. RAR collaborated in the design of the study, reviewed and edited the protocol. All authors read and approved the final manuscript.

## Supplementary Material

Additional file 1 **Search strategy for electronic databases.** Search strategy developed for CENTRAL, MEDLINE (OVID) and EMBASE (OVID). Click here for file
