# Supplementary material for: Effects of lifestyle modification after breast cancer treatment: a systematic review protocol
Source: Syst Rev. 2014 Jul 5;3:72. doi: 10.1186/2046-4053-3-72 (PMC4107599; doi:10.1186/2046-4053-3-72)
Supplement: Additional file 1 — Search strategy for electronic databases. Search strategy developed for CENTRAL, MEDLINE (OVID) and EMBASE (OVID). [file 2046-4053-3-72-S1.pdf]

Additional File – Search strategy for electronic databases

CENTRAL

#1 MeSH descriptor: [Breast Neoplasms] explode all trees
#2 breast near cancer*
#3 breast near neoplasm*
#4 breast near carcinom*
#5 breast near tumour*
#6 breast near tumor*
#7 breast near malignan*
#8 #1 or #2 or #3 or #4 or #5 or #6 or #7
#9 MeSH descriptor: [Health Behavior] explode all trees
#10 MeSH descriptor: [Health Promotion] explode all trees
#11 MeSH descriptor: [Exercise] explode all trees
#12 MeSH descriptor: [Exercise Therapy] explode all trees
#13 MeSH descriptor: [Sports] explode all trees
#14 MeSH descriptor: [Physical Fitness] explode all trees
#15 MeSH descriptor: [Diet Therapy] explode all trees
#16 MeSH descriptor: [Feeding Behavior] explode all trees
#17 MeSH descriptor: [Diet] explode all trees
#18 MeSH descriptor: [Physical Education and Training] explode all trees
#19 MeSH descriptor: [Life Style] explode all trees
#20 MeSH descriptor: [Health Education] explode all trees
#21 #9 or #10 or #11 or #12 or #13 or #14 or #15 or #16 or #17 or #18 or #19 or #20
#22 (lifestyl* or life styl*):ti,ab,kw
#23 (health* near (behav* or educ* or promot*)):ti,ab,kw
#24 (exercis* or physic* activit* or exert* or physic* fit* or sport*):ti,ab,kw
#25 (walk* or jog* or swim* or bicyc* or cycling or weight lift* or gymnastic or danc*):ti,ab,kw
#26 ((strength or resistance or circuit or enduran* or aerob* or physic* or fit*) next train*):ti,ab,kw
#27 (nutri* or diet*):ti,ab,kw
#28 #22 or #23 or #24 or #25 or #26 or #27
#29 #21 or #27
#30 #8 AND #29

MEDLINE (OVID)

#1 exp breast neoplasms/
#2 exp breast/
#3 breast.tw.
#4 2 or 3
#5 exp neoplasms/
#6 exp lymphedema/

#7 exp radiotherapy/
#8 or/5-7
#9 4 and 8
#10 (breast adj25 neoplasm$).tw,ot.
#11 (breast adj25 cancer$).tw,ot.
#12 (breast adj25 tumour$).tw,ot.
#13 (breast adj25 tumor$).tw,ot.
#14 (breast adj25 carcinoma$).tw,ot.
#15 (breast adj25 adenocarcinoma$).tw,ot.
#16 (breast adj25 ductal).tw,ot.
#17 (breast adj25 infiltrating).tw,ot.
#18 (breast adj25 lobular).tw,ot.
#19 (breast adj25 medullary).tw,ot.
#20 exp mammary neoplasms/
#21 (mammary adj25 neoplasm$).tw,ot.
#22 (mammary adj25 cancer$).tw,ot.
#23 (mammary adj25 tumour$).tw,ot.
#24 (mammary adj25 tumor$).tw,ot.
#25 (mammary adj25 carcinoma$).tw,ot.
#26 (mammary adj25 adenocarcinoma$).tw,ot.
#27 (mammary adj25 ductal).tw,ot.
#28 (mammary adj25 infiltrating).tw,ot.
#29 (mammary adj25 lobular).tw,ot.
#30 (mammary adj25 medullary).tw,ot.
#31 exp mastectomy/
#32 or/10-31
#33 1 or 9 or 32
#34 exp Health Behavior/
#35 exp Health Promotion/
#36 exp exercise/
#37 exp exercise therapy/
#38 exp Sports/
#39 exp Physical Fitness/
#40 exp Diet Therapy/
#41 exp Feeding Behavior/
#42 exp Diet/
#43 exp "Physical Education and Training"/
#44 exp Life Style/
#45 exp Health Education/
#46 (lifestyl$ or life styl$).tw,ot.
#47 (health$ adj6 (behav$ or educ$ or promot$)).tw,ot.
#48 (exercis$ or physic$ activit$ or exert$ or physic$ fit$ or sport$).tw,ot.
#49 (walk$ or jog$ or swim$ or bicyc$ or cycling or weight lift$ or gymnastic or danc$).tw,ot.
#50 ((strength or resistance or circuit or enduran$ or aerob$ or physic$ or fit$) adj6 train$).tw,ot.
#51 (nutri$ or diet$).tw,ot.
#52 or/34-51

#53 Randomized Controlled Trials as Topic/
#54 randomized controlled trial/
#55 Random Allocation/
#56 Double Blind Method/
#57 Single Blind Method/
#58 clinical trial/
#59 clinical trial, phase i.pt
#60 clinical trial, phase ii.pt
#61 clinical trial, phase iii.pt
#62 clinical trial, phase iv.pt
#63 controlled clinical trial.pt
#64 randomized controlled trial.pt
#65 multicenter study.pt
#66 clinical trial.pt
#67 exp Clinical Trials as topic/
#68 (clinical adj trial$).tw
#69 ((singl$ or doubl$ or treb$ or tripl$) adj (blind$3 or mask$3)).tw
#70 PLACEBOS/
#71 placebo$.tw
#72 randomly allocated.tw
#73 (allocated adj2 random$).tw
#74 or/53-73
#75 case report.tw
#76 letter/
#77 historical article/
#78 or/75-77
#79 74 not 78
#80 33 and 52 and 79

EMBASE (OVID)

#1 exp breast neoplasms/
#2 exp breast/
#3 breast.tw.
#4 2 or 3
#5 exp neoplasms/
#6 exp lymphedema/
#7 exp radiotherapy/
#8 or/5-7
#9 4 and 8
#10 (breast adj25 neoplasm$).tw,ot.
#11 (breast adj25 cancer$).tw,ot.
#12 (breast adj25 tumour$).tw,ot.
#13 (breast adj25 tumor$).tw,ot.
#14 (breast adj25 carcinoma$).tw,ot.

#15 (breast adj25 adenocarcinoma$).tw,ot.
#16 (breast adj25 ductal).tw,ot.
#17 (breast adj25 infiltrating).tw,ot.
#18 (breast adj25 lobular).tw,ot.
#19 (breast adj25 medullary).tw,ot.
#20 exp mammary neoplasms/
#21 (mammary adj25 neoplasm$).tw,ot.
#22 (mammary adj25 cancer$).tw,ot.
#23 (mammary adj25 tumour$).tw,ot.
#24 (mammary adj25 tumor$).tw,ot.
#25 (mammary adj25 carcinoma$).tw,ot.
#26 (mammary adj25 adenocarcinoma$).tw,ot.
#27 (mammary adj25 ductal).tw,ot.
#28 (mammary adj25 infiltrating).tw,ot.
#29 (mammary adj25 lobular).tw,ot.
#30 (mammary adj25 medullary).tw,ot.
#31 exp mastectomy/
#32 or/10-31
#33 1 or 9 or 32
#34 exp Health Behavior/
#35 exp Health Promotion/
#36 exp Exertion/
#37 exp exercise/
#38 exp exercise therapy/
#39 exp Sports/
#40 exp Physical Fitness/
#41 exp Diet Therapy/
#42 exp Feeding Behavior/
#43 exp Diet/
#44 exp "Physical Education and Training"/
#45 exp Life Style/
#46 exp Health Education/
#47 (lifestyl$ or life styl$).tw,ot.
#48 (health$ adj6 (behav$ or educ$ or promot$)).tw,ot.
#49 (exercis$ or physic$ activit$ or exert$ or physic$ fit$ or sport$).tw,ot.
#50 (walk$ or jog$ or swim$ or bicyc$ or cycling or weight lift$ or gymnastic or danc$).tw,ot.
#51 ((strength or resistance or circuit or enduran$ or aerob$ or physic$ or fit$) adj6 train$).tw,ot.
#52 (nutri$ or diet$).tw,ot.
#53 or/34-52
#54 Clinical trial/
#55 Randomized controlled trial/
#56 Randomization/
#57 Single blind procedure/
#58 Double blind procedure/
#59 Crossover procedure/
#60 Placebo/

#61 Randomi?ed controlled trial$.tw.
#62 Rct.tw.
#63 Random allocation.tw.
#64 Randomly allocated.tw.
#65 Allocated randomly.tw.
#66 (allocated adj2 random).tw.
#67 Single blind$.tw.
#68 Double blind$.tw.
#69 ((treble or triple) adj (blind$)).tw.
#70 Placebo$.tw.
#71 Prospective study/
#72 Or/54-71
#73 Case study/
#74 Case report.tw.
#75 Abstract report/
#76 letter/
#77 Or/73-76
#78 72 not 77
#79 32 and 53 and 78
